# The association of mannose-binding lectin 2 polymorphisms with outcome in very low birth weight infants

**DOI:** 10.1371/journal.pone.0178032

**Published:** 2017-05-30

**Authors:** Annika Hartz, Julia Pagel, Alexander Humberg, Michael Preuss, Lena Schreiter, Jan Rupp, Julia Figge, Christian M. Karsten, Peter Nürnberg, Egbert Herting, Wolfgang Göpel, Christoph Härtel, for the German Neonatal Network (GNN)

**Affiliations:** 1 Department of Pediatrics, University Hospital Lübeck, Lübeck, Germany; 2 Institute of Systemic Inflammation Research, University Hospital Lübeck, Lübeck, Germany; 3 Department of Infectious Diseases and Microbiology, University Hospital Lübeck, Lübeck, Germany; 4 Institute for Medical Biometry and Statistics, University of Lübeck, Lübeck, Germany; 5 Cologne Center for Genomics, Cologne, Germany; Centre Hospitalier Universitaire Vaudois, FRANCE

## Abstract

**Objectives:**

Studies on the influence of mannose-binding lectin (MBL) deficiency on infection susceptibility in preterm infants have yielded controversial results. We investigated the association of genotype-based MBL levels with outcome in very-low-birth weight infants (VLBWI).

**Methods:**

We genotyped 3 genetic variants of MBL2 (rs1800450, rs1800451, rs5030737) in 6878 VLBWI. MBL plasma levels were categorized as normal (wild type, A/A), low (heterozygotes, A/O) or undetectable (homozygotes, O/O). Primary outcome was the effect of genotype-based MBL2 levels on blood-culture proven and clinical sepsis during primary stay in hospital. We also evaluated burden of infection within 24 months after discharge.

**Results:**

We found no association between MBL levels and sepsis risk in the whole cohort. Infants without measurable MBL levels born between 32 0/7 to 36 6/7 weeks of gestation, however, had a higher rate of Gram-negative sepsis than infants with normal or reduced MBL levels. In a follow-up investigation at 24 months (n = 1070 infants), infants without measurable MBL levels suffered more frequently from stomatitis and urinary tract infection.

**Conclusions:**

In a large cohort of VLBWI MBL2 deficiency had no major impact on infection risk unless children were born between 32 0/7 and 36 6/7 weeks of gestation.

## Introduction

Infections are a major threat to newborn infants, especially to those who are born preterm. The infection risk profile of preterm infants is mainly influenced by gestational age, birth weight and environmental exposures. Additionally, genetic risk factors for infection are proposed to play a crucial role, however, have not been confirmed yet in highly susceptible preterm infants.

One potential candidate is the gene encoding for mannose-binding lectin (MBL), a collagenous lectin (collectin) and pattern recognition molecule initiating the lectin pathway of complement. MBL binds to membrane polysaccharides of bacteria, viruses and a variety of other pathogens and mediates opsonization and phagocytosis. It also mediates inflammatory cytokine production by acting as a Toll-like receptor (TLR)-2/-6 co-receptor [1]. Several genetic variants of exon 1 of the MBL2 gene lead to MBL deficiency, i.e. single nucleotide polymorphisms (SNPs) in codon 52 (Arg to Cys, allele D), codon 54 (Gly to Asp, allele B) and codon 57 (Gly to Glu, allele C). Heterozygote carriers in any of these SNPs are subsumed under the designation A/O and show 5- to 10-fold reduced amounts of functional MBL as compared to wildtype individuals (A/A) while undetectable levels of MBL are noted in homozygous carriers of any of the variants (O/O) [2]. Low or undetectable MBL levels were found to be associated with an increased susceptibility to infectious diseases, especially in children and immune-compromised individuals [3, 4]. In preterm infants, results of genotype-based studies are controversial with regard to infection risk and adverse long-term outcome. This is mainly due to different cohort sizes (including 47–1832 preterm and term infants) [5], variable outcome definitions and the fact that MBL levels also correlate with gestational age independent of the genetic background [5–7].

We performed a large-scale study to determine the effect of MBL2 polymorphisms on short-term outcome of well phenotyped very-low-birth-weight infants (VLBWI) enrolled in the German Neonatal Network. We also aimed to assess the burden of infections within the first 24 months of life according to genotype-based MBL levels.

## Materials and methods

### Design, setting and subjects

The German Neonatal Network (GNN) is a population-based cohort study enrolling VLBWI at 54 neonatal intensive care units in Germany. The study period was January 2009 until December 2014 (inclusion criteria: birth weight < 1500 g and gestational age ≤ 36 6/7 weeks). After obtaining written informed consent from the parents, a DNA-sample of the infant was collected via buccal swab and/or cord tissue and transferred to the study center (University of Lübeck). Data regarding antenatal and postnatal treatment and outcome were recorded by according data sheets at the participating centers. After discharge, data sheets were sent to the study center. Data quality was evaluated by a physician trained in neonatology via annual on-site monitoring of completed data sets. After monitoring, data were coded and linked to the according infant DNA-sample by number.

For the 24 months follow-up, parents of surviving infants received a questionnaire (according to KiGGS survey 1–2 years from Robert Koch Institute, Germany [8]) which included the frequency of episodes of infections after discharge from the primary stay in hospital. The results were also linked to the infants DNA-sample by number.

### Genotyping

DNA extraction was performed using a commercial DNA purification kit (Qiagen, Hilden, Germany). The DNA was washed twice and eluted. Genotypes were determined with the TaqMan 5′ nuclease assay (Applied Biosystems, Foster City, CA) and the 7900HT Real-Time PCR System. Context sequences were as follows:

rs1800450: 5’TGGTTCCCCCTTTTCTCCCTTGGTG[C/T]CATCACGCCCATCTTTGCCTGGGAA3’

rs1800451: 5’ACACGTACCTGGTTCCCCCTTTTCT[C/T]CCTTGGTGCCATCACGCCCATCTTT 3’

rs5030737: 5’CCCTTTTCTCCCTTGGTGCCATCAC[A/G]CCCATCTTTGCCTGGGAAGCCGTTG 3’

The distribution of genotypes was appropriate to allele frequencies, as determined by Hardy-Weinberg equilibrium.

### Genome Wide Association Study (GWAS)

DNA was isolated from umbilical cord samples with Gentra Puregene Blood Kit (Qiagen, Hilden, Germany) and analysed at the Cologne Center for Genomics (CCG) using the Axiom^®^ Genome-Wide CEU 1 Array Plate (Affymetrix, Santa Clara, CA, USA). Genotype assessment was based on imputation.

### MBL2 genotype categories

MBL plasma levels were categorized as being normal in individuals with no variants (A/A), low in individuals heterozygous for one or more variant alleles (A/O) and undetectable for those homozygous for one or more variants (O/O).

### Definition of primary outcome and power analysis

Primary outcome measure was risk of blood culture proven sepsis, defined as at least two clinical signs of sepsis (temperature > 38°C or < 36.5°C, tachycardia > 200/min, new onset or increased frequency of bradycardias or apneas, hyperglycemia > 140 mg/dl, base excess < -10 mval/l, changed skin color, increased oxygen requirements) and one laboratory sign (C-reactive protein > 2 mg/dl, immature/neutrophil ratio > 0.2, white blood cell count < 5/nl, platelet count < 100/nl) and proven pathogen in the blood culture. Co-primary outcome was clinical sepsis without evidence of pathogen in the blood culture, but clinical and laboratory signs of sepsis and treatment with antibiotics for at least 5 days.

The cohort size was based on the effect size reported by Swierzko et al. [9] who found a 7.6% infection risk in AA + AO (118 affected, 1431 controls) vs. 12.1% infection risk in AO + OO (30 affected, 221 controls). In order to achieve a power of 0.90 with a level of significance of 0.05 the required cohort size consists of 3475 infants. Definitions of secondary outcome measures are described in supporting information S1 File.

### Statistical analysis

Data analysis was performed using the SPSS 22.0 data analysis package (IBM, Munich, Germany). Hypotheses were evaluated in univariate analyses with Fisher´s exact test, Mann-Whitney U-test and Kruskal-Wallis test. A *p* value < 0.05 was considered as statistically significant for single tests.

### Ethics

The study parts were approved by the local committee (votum no. 08–022) on research in human subjects of the University of Lübeck and the local ethical committees at the other study centers, specifically: Ethical Board of the Medical Chamber of the North Rhine region, Ethical Board of the University of Aachen, Ethical Board of the University of Bonn, Ethical Board of the Medical Chamber of the federal state of Mecklenburg-Vorpommern, Ethical Board of the Medical Chamber of Berlin, Ethical Board of the University of Magdeburg, Ethical Board of the University of Halle, Ethical Board of the University of Tübingen, Ethical Board of the Medical School Hannover, Ethical Board of the University of Cologne, Ethical Board of the University of Essen, Ethical Board of the Medical Chamber of the Westphalia-Lippe region, Ethical Board of the Medical Chamber of Hamburg, Ethical Board of the Medical Chamber of the federal state of Hessen, Ethical Board of the Medical Chamber of the federal state of Baden-Württemberg, Ethical Board of the Medical Chamber of the federal state of Bavaria, Ethical Board of the Saar University.

## Results

### Genotype frequencies

We genotyped 7747 infants for rs1800450, 6775 infants for rs1800451 and 6905 infants for rs5030737. As the frequency of MBL genotypes varies greatly between different ethnic groups, we restricted our analysis of genotype-based MBL levels to infants with European background (n = 6878). The incidence of the variant genotype rs1800450 was 25.6% (23.8% heterozygous, 1.8% homozygous), the incidence of rs1800541 was 3.7% (3.6% heterozygous, 0.1% homozygous). For rs5030737 the incidence was 12.7% (12.2% heterozygous and 0.5% homozygous) (Table 1).

**Table 1:** Frequency of MBL2 polymorphisms in the European cohort and number of genotyped infants (PCR + Genome-Wide Association Study).

	Localization in exon 1	A/A	A/O	O/O	total
rs1800450 (B allele)	-54 G → D	5761 (74.4%)	1846 (23.8%)	140 (1.8%)	7747
rs1800451 (C allele)	-57 G → E	6523 (96.3%)	247 (3.6%)	5 (0.1%)	6775
rs5030737 (D allele)	-52 R → C	6028 (87.3%)	842 (12.2%)	35 (0.5%)	6905

Genotype-based MBL levels were normal in 57.2% (n = 3929), low in 40.3% (n = 2769) and not measurable in 2.6% of infants (n = 180). There were no differences in clinical data between groups according to genotype-based MBL levels, apart from a slightly lower gestational age in infants without measurable MBL levels (Table 2).

### Effect of MBL2 polymorphisms on the primary outcome

As shown in Table 2, genotype-based MBL levels were not associated with risk for blood-culture proven or clinical sepsis. MBL2 variants also showed no association with the risk of inflammation-mediated disorders such as surgical necrotizing enterocolitis (NEC) or bronchopulmonary dysplasia (BPD). We noted a higher rate of intracerebral hemorrhage (ICH) grade I and a reduced rate of higher grade ICH in infants without measurable MBL levels as compared to infants with normal MBL levels.

**Table 2:** Clinical characteristics of VLBW cohort according to genotype-based MBL levels.

MBL levels	Normal	Low	Not measurable	p
**Number of infants, %**	3929, 57.2	2769, 40.3	180, 2.6	
**Gestational age (mean ± SD, weeks)**	28.7 (2.7)	28.8 (2.7)	28.6 (2.6)	0.04*
**Birth weight (mean ± SD, g)**	1057 (307)	1073 (299)	1063 (301)	0.1*
**Male gender (%)**	52.1	52.0	42.8	0.05
**Multiples (%)**	33.0	35.0	38.0	0.1
**Small for gestational age (%)**	18.6	17.7	11.7	0.05
**Antibiotic therapy (%)**	83.3	83.7	83.7	0.9
**Clinical sepsis (%)**	28.5	28.5	27.2	0.9
**Blood culture proven sepsis (%)**	12.0	11.3	12.9	0.6
Late-onset sepsis	11.5	10.5	11.8	0.4
Gram-negative sepsis	2.3	2.4	1.7	0.8
Gram-positive sepsis	9.7	9.4	11.2	0.7
Gram-positive sepsis (without CoNS)	2.3	2.3	1.7	0.9
Sepsis in cases of non-survivors (%)	6.5	7.2	13.0	0.5
**Intracerebral hemorrhage (ICH, %)**	16.8	17.2	15.6	0.5
				0.03
Grade I	6.3	7.4	10.5	
Grade II	4.0	4.6	5.3	
Grade III	3.3	2.5	0.7	
Grade IV	3.0	2.6	1.3	
**Periventricular leukomalacia (PVL, %)**	3.4	2.8	2.0	0.2
**Surgery for NEC (%)**	2.2	2.1	2.3	0.9
**Surgery for FIP (%)**	2.1	1.9	0.6	0.3
Surgery for NEC or FIP (%)	4.2	3.9	2.9	0.6
NEC/FIP in cases of non-survivors (%)	15.0	10.4	40.0	0.1
**Severe complication (%)**	14.3	13.1	10.6	0.1
**BPD (%)**	13.5	12.6	10.4	0.3
**Death (%)**	3.3	2.8	3.9	0.5

Severe complication: ICH grade III or IV, PVL, surgery for necrotizing enterocolitis (NEC) or focal intestinal perforation (FIP), treatment for retinopathy of prematurity (ROP) or death; CoNS: coagulase-negative staphylococci;

p-values are derived from Fisher´s exact test or Kruskal-Wallis-test if indicated (*).

To take into account that MBL plasma levels increase with gestational age, we performed subgroup analyses in infants born 22 0/7-27 6/7, 28 0/7-31 6/7 and 32 0/7-36 6/7 weeks of gestation. Sepsis-related outcomes were not associated with genotype-based MBL levels in infants born < 32 weeks of gestation (Tables 3 and 4). Infants without measurable MBL levels born between 32 0/7 to 36 6/7 weeks of gestation, however, did have a higher rate of Gram-negative sepsis as compared to infants with normal MBL levels or reduced MBL levels (5.9% vs. 0.4%/0%, p = 0.001, Table 5).

**Table 3:** Infection risk according to MBL levels in gestational age group 22 0/7–27 6/7 weeks of gestation.

MBL levels	Normal	Low	Not measurable	p
**Number of infants**	1524	991	74	
**Clinical sepsis (%)**	45.8	46.5	46.7	0.7
**Blood culture proven sepsis (%)**	19.9	20.3	20.3	0.8
Late-onset sepsis	19.2	18.9	18.9	0.9
Gram-negative sepsis	4.3	4.7	1.4	0.3
Gram-positive sepsis	15.7	16.6	18.9	0.7
Gram-positive sepsis (without CoNS)	3.7	3.9	4.1	0.8

CoNS: coagulase-negative staphylococci; p-values are derived from Fisher´s exact test.

**Table 4:** Infection risk according to MBL levels in gestational age group 28 0/7–31 6/7 weeks of gestation.

MBL levels	Normal	Low	Not measurable	p
**Number of infants**	1876	1406	87	
**Clinical sepsis (%)**	19.4	20.5	12.5	0.2
**Blood culture proven sepsis (%)**	7.8	6.8	6.9	0.3
Late-onset sepsis	7.4	6.3	5.7	0.2
Gram-negative sepsis	1.2	1.2	1.1	0.9
Gram-positive sepsis	6.7	5.9	5.7	0.6
Gram-positive sepsis (without CoNS)	1.5	1.4	0	0.5

CoNS: coagulase-negative staphylococci; p-values are derived from Fisher´s exact test.

**Table 5:** Infection risk according to MBL levels in gestational age group 32 0/7–36 6/7 weeks of gestation.

MBL levels	Normal	Low	Not measurable	p
**Number of infants**	465	325	17	
**Clinical sepsis (%)**	8.5	7.6	17.6	0.3
**Blood culture proven sepsis (%)**	3.0	2.5	11.8	0.09
Late-onset sepsis	3.0	2.2	11.8	0.06
Gram-negative sepsis	0.4	0	5.9	0.001
Gram-positive sepsis	2.6	2.5	5.9	0.7
Gram-positive sepsis (without CoNS)	0.6	0.6	0	0.9

CoNS: coagulase-negative staphylococci; p-values are derived from Fisher´s exact test.

### Association of MBL2 polymorphisms with infection rate in the first 24 months of life

In a follow-up investigation, parents of GNN infants were asked for the number of infection episodes via questionnaire at the age of 24 months. In a representative cohort of n = 1070 infants we found no association of MBL genotypes with the incidence of common cold, tonsillitis, bronchitis, croup, bacterial conjunctivitis and local fungal infections reported by parents (Table 6).

**Table 6:** Episodes of infections in the first 24 months of life according to genotype-based MBL levels.

MBL levels	Normal n = 593 infants	Low n = 452 infants	Not measurable n = 25 infants	p1	p2
**Cold**	3.43 (2.7)	3.62 (5.3)	3.56 (2.5)	0.7	0.6
**Tonsillitis**	0.21 (0.7)	0.21 (0.7)	0.06 (0.2)	0.3	0.3
**Bronchitis**	1.12 (2.0)	1.12 (1.7)	1.30 (2.1)	0.9	0.9
**Croup**	0.18 (0.7)	0.12 (0.4)	0.05 (0.2)	0.4	0.5
**Gastroenteritis**	0.85 (1.0)	1.03 (2.7)	1.05 (1.5)	0.9	0.9
**Conjunctivitis**	0.43 (0.9)	0.50 (1.1)	0.21 (0.4)	0.4	0.4
**Local fungal infection**	0.34 (1.0)	0.41 (0.9)	0.47 (0.9)	0.2	0.5
**Herpes stomatitis**	0.08 (0.3)	0.09 (0.3)	0.8 (1.9)	0.006	0.02
**Urinary tract infection**	0.04 (0.2)	0.04 (0.2)	0.33 (0.7)	0.007	0.006

Given is the number of episodes as mean (SD). p1 = Normal MBL level vs. Not measurable MBL level; p2 = Low MBL level vs. Not measurable MBL level (Mann-Whitney U-test)

**Fig 1:**
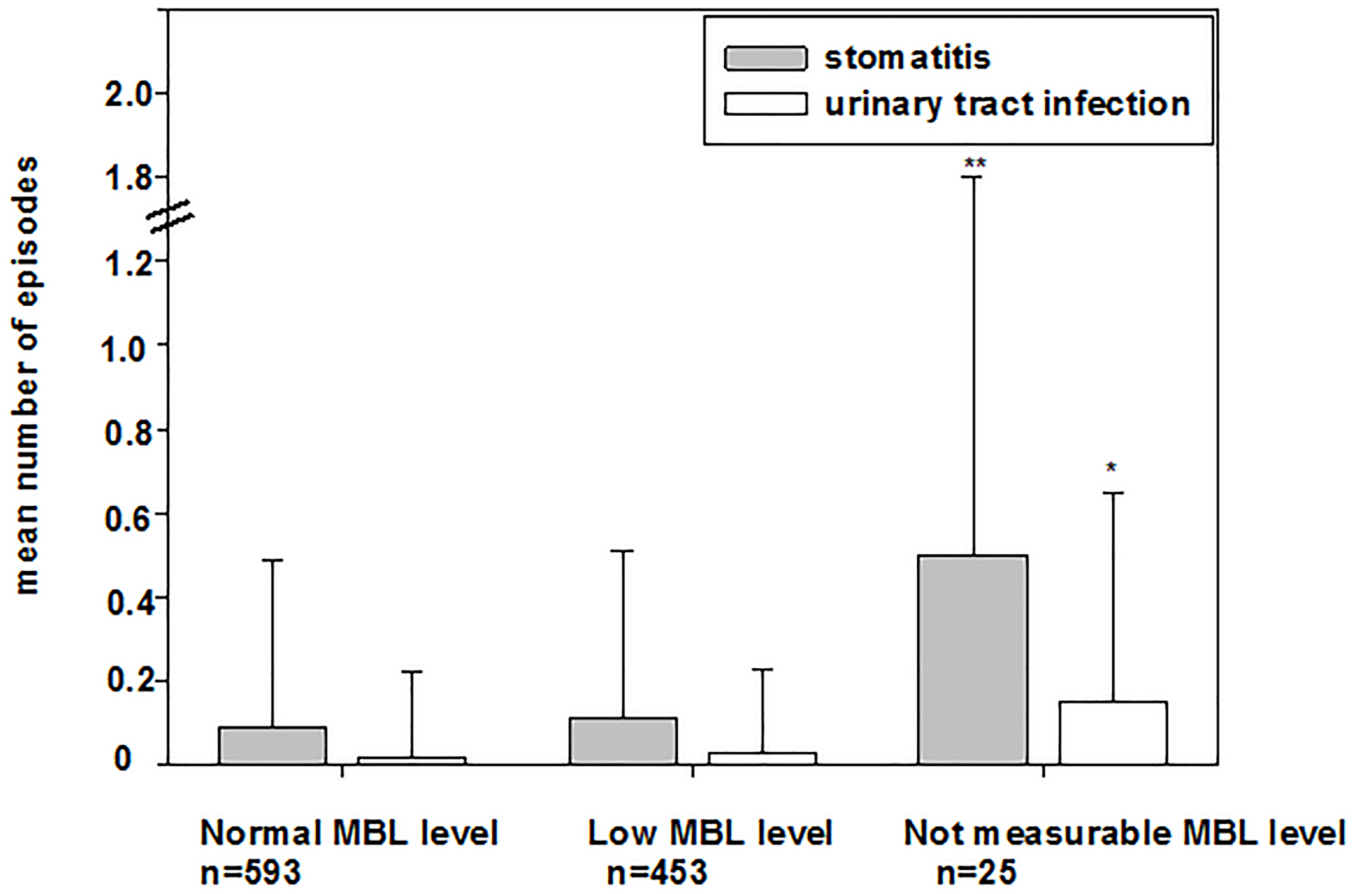
Episodes of herpes stomatitis and bacterial urinary tract infection in the first 24 months of life. The mean±SD number of episodes of episodes of stomatitis and urinary tract infection (UTI) are based on parents’ responses to the KIGGS questionnaire at 24 months of age. Data are described according to genotype-based MBL levels. Infants without measurable MBL levels had a higher rate of herpes stomatitis as compared to infants with normal MBL levels (p = 0.004) and low MBL levels (p = 0.02) and a higher frequency of bacterial UTI as compared to infants with normal MBL levels (p = 0.03, Mann-Whitney U-test).

Infants without measurable MBL levels suffered more frequently from stomatitis and urinary tract infection than infants with normal/low MBL levels (p^1^ normal vs. not measurable, p^2^ low vs. not measurable; stomatitis: p^1^ = 0.006, p^2^ = 0.02, urinary tract infection: p^1^ = 0.007, p^2^ = 0.006, Table 6, Fig 1).

## Discussion

We performed a large-scale study to address the association of genetic polymorphisms in exon 1 of the MBL2 gene with infection risk in European VLBWI. We found no association between genotype-based MBL levels and blood-culture proven or clinical sepsis risk in the whole cohort of VLBWI. There appears to be an association, however, between non-measurable MBL levels and Gram-negative sepsis risk in the subgroup of infants born between 32 0/7 to 36 6/7 weeks of gestation. Genotype-based MBL levels were not associated with major infection risk within the first 24 months after discharge, apart from a higher risk for herpes stomatitis and bacterial UTI in infants without measurable MBL levels.

One potential explanation for our results is that MBL levels are gestational age dependent, regardless of genotype. MBL levels of term infants have been shown to be similar to adult levels while ranging between 36% and 64% thereof in preterm infants (reviewed in [10]). One could hypothesize that the genotype-based differences only become relevant during immunological maturation. As such, MBL levels of AA individuals increase with age, while MBL levels of AO or OO individuals remain low or undetectable [11]. Thus, a significant effect of the polymorphisms could not be expected in very preterm infants, which are the majority of our cohort, while in the subgroup of infants with higher gestational age (32 0/7 to 36 6/7 weeks of gestation) the difference in MBL plasma levels might contribute to Gram-negative sepsis risk. Likewise, infants with variant MBL2 genotype would carry an increased risk for infection during early childhood. We found a higher frequency of urinary tract infection and herpes stomatitis in infants without measurable MBL2 levels at 24 months-follow-up. This is in line with previous observations demonstrating a role for MBL deficiency for *Herpes simplex virus 2* infections [12]. The number of episodes of other relevant infections such as bronchitis or gastroenteritis was not influenced by genotype-based MBL levels. A different binding affinity of MBL to different pathogens (reviewed in [1]) may be a reason for this. As only a minority of infections appears influenced, we postulate that genotype-based MBL2 levels only play a minor role in the complex situation of immunological maturation of preterm infants.

MBL has been shown to contribute to blood coagulation. For example, MBL-deficient mice were found to have a higher risk of developing disseminated intravascular coagulation (DIC) in case of sepsis [13]. Interestingly, we found a potential protective effect of non-measurable MBL levels on the severity of intracerebral hemorrhage. This may seem surprising at first, but unlike the DIC in sepsis, the cerebral hemorrhage in preterm neonates is not primarily caused by infection but rather by changes in oxygenation and circulation. The protective effect might be explained by reduced binding of MBL to damaged tissue and consecutively decreased complement activation and production of inflammatory cytokines. This theory is supported by animal reperfusion models demonstrating that MBL-deficient mice develop significantly less tissue damage in heart, gut and kidney (reviewed in [14]).

The major strengths of our study are the large cohort size and detailed phenotypic characterization of VLBWI. However, our design has limitations. Genotype-phenotype correlations might be influenced by other SNPs in the gene which were not examined in our study. For example, Steffensen et al. [15] showed that SNPs in the promoter region of the MBL2 gene also affect the MBL plasma levels, with the LX promoter allele having as strong an effect on plasma MBL as the exon 1 mutations. Furthermore, we did not measure MBL levels directly in plasma of VLBWI.

## Conclusions

The investigation of a large cohort of VLBWI did not confirm an association between genotype-based MBL2 levels and the risk for infection or other relevant short term outcomes in this group, unless children were born were born with relatively high gestational age (32 0/7 to 36 6/7 weeks of gestation). A correlation of genotype-based MBL levels with infection frequency in a 24 months-follow-up could only be seen for specific diseases, a general increase in infection frequency in case of low or undetectable MBL levels was not found.

## Supporting information

S1 File Definition of secondary outcome measures. (DOCX)
