# Supplementary material for: The association of mannose-binding lectin 2 polymorphisms with outcome in very low birth weight infants
Source: PLoS One. 2017 May 30;12(5):e0178032. doi: 10.1371/journal.pone.0178032 (PMC5448758; doi:10.1371/journal.pone.0178032)
Supplement: S1 File — (DOCX) [file pone.0178032.s001.docx]

**Supporting Information**

**Definition of secondary outcome measures**

**Antibiotic therapy** is defined as any antibiotic therapy independent of substance or the duration of treatment.

**Late onset sepsis** was defined as blood culture proven sepsis with onset after 72 h of life.

**Gram-negative sepsis or Gram-positive sepsis** were defined as blood-culture proven sepsis with evidence of Gram-negative or any Gram-positive bacteria in the blood culture, respectively.

**Gram-positive sepsis (without CoNS)** excluded cases with proof of coagulase-negative Staphylococci in blood culture from the group of Gram-positive sepsis.

**Sepsis in cases of non-survivors** is the proportion of death before discharge in the population of infants who suffered from or blood culture proven or clinical sepsis, respectively.

**NEC requiring surgery** was defined as clinical necrotizing enterocolitis (NEC) classified as Bell Stage II or Bell Stage III with need for peritoneal drainage, laparotomy with or without resection of necrotic gut, and macroscopic diagnosis of NEC made by the attending surgeon.

**FIP requiring surgery** was defined as occurrence of spontaneous intestinal perforation with need for peritoneal drainage or laparotomy and macroscopic confirmation of isolated focal intestinal perforations (FIPs) (without inflammatory component) rather than NEC made by the attending surgeon.

**NEC or FIP requiring surgery** is the combined outcome with respect to the main burden of surgery due to major abdominal complications after preterm birth.

**NEC/FIP in cases of non-survivors** is the proportion of death before discharge in the population of infants who suffered from NEC or FIP requiring surgery.

**Intracerebral hemorrhage (ICH) grades I-IV** were diagnosed according to the ultrasound criteria of Papile.

**Cystic periventricular leukomalacia (PVL)** was defined as periventricular lesions. **Bronchopulmonary dysplasia (BPD)** was diagnosed when needing supplemental oxygen or assisted ventilation evaluated at 36 weeks of post menstrual age.

**Severe complication** was defined as diagnosis of at least one of the following outcome measures: ICH grade III or intracerebral parenchymal hemorrhage, PVL, retinopathy of prematurity (ROP) requiring surgery, NEC or FIP requiring surgery or need for ventriculoperitoneal shunting.

**Death** was defined as death occurring after admission to neonatal intensive care unit within the primary stay in hospital.
